# University students in the era of climate change: climate distress is associated with academic burnout and engagement through academic amotivation

**DOI:** 10.3389/fpsyg.2026.1878132

**Published:** 2026-08-26

**Authors:** Luciano Romano, Claudia Russo, Davide Clemente, Vanessa Marchetti, Elena Rinallo, Caterina Fiorilli, Maria Ojala, Angelo Panno

**Affiliations:** 1 Experimental and Applied Psychology Laboratory, Department of Health and Life Sciences, Università Europea di Roma, Rome, Italy; 2 Department of Human Sciences, LUMSA University, Rome, Italy; 3 Department of Education Sciences, University of Roma Tre, Rome, Italy; 4 Frontiers of Arctic and Global Resilience (FRONT) and Teacher Education, School and Society (TESS), Faculty of Education and Psychology, University of Oulu, Oulu, Finland; 5 School of Behavioural, Social and Legal Sciences, Örebro University, Örebro, Sweden

**Keywords:** academic amotivation, academic burnout, academic engagement, climate distress, university students

## Abstract

**Introduction:**

Climate change has measurable impacts on the psycho-physical health of individuals, with disproportionate effects on current and future youth cohorts. Many young individuals experience climate distress, characterized by negative emotions and thoughts related to climate change that can impact their daily lives. Although previous studies have documented how climate change has increased concerns regarding academic careers, no research has explored how climate distress may compromise students’ adjustment in terms of burnout and engagement. Furthermore, no studies have examined the motivational processes that could explain this relationship. Therefore, the present study examined, within a unified model, the relationships among climate distress, engagement, and burnout, hypothesizing that these relationships occur through academic amotivation. Specifically, it was hypothesized that climate distress would be associated with increased academic burnout and decreased engagement through academic amotivation.

**Methods:**

A convenience sample of 375 university students, aged 18–37 years (*M* = 21.64, SD = 2.28), was involved, and a path analysis model was tested to verify the formulated hypotheses.

**Results:**

The results confirmed the indirect association between climate distress and both engagement and academic burnout through academic amotivation. Specifically, climate distress was associated with increased academic amotivation, which in turn, was associated with lower engagement and higher burnout.

**Discussion:**

The findings highlight an association between climate distress and academic maladjustment among university students, underscoring the pivotal role of academic amotivation. The results indicate the importance of addressing climate distress in academic contexts considering the negative repercussions that may affect students’ wellbeing and academic career trajectories.

## 1 Introduction

The academic literature extensively demonstrates that climate change entails profoundly negative consequences for individuals. While numerous studies (e.g., Rocque et al., 2021; Abbass et al., 2022) have focused on the direct and indirect adverse effects of large-scale climate change (e.g., greenhouse effect, extreme weather events, climate migration, ocean warming), mounting evidence reveals equally significant consequences in terms of psychological and physical wellbeing. Several studies have shown strong associations between climate change and individuals’ mental health (e.g., Cianconi et al., 2020; Clayton, 2020; Hwong et al., 2022).

### 1.1 Climate change impacts on young people

In a global context where the Intergovernmental Panel on Climate Change projects a high probability of global warming reaching 1.5 °C even under low greenhouse gas scenarios (IPCC, 2023), the individuals most affected by the material and psycho-physical consequences of climate change will be young people from the current and from future generations. As highlighted by recent findings, young people experience intense feelings of concern, insecurity, distress, and more severe consequences related to climate change, such as eco-anxiety, PTSD, and depressive symptoms (e.g., Manning and Clayton, 2018; Clemens et al., 2022; Gislason et al., 2021). These findings have led numerous researchers to develop specific educational plans within educational institutions, with the aim of promoting pedagogy grounded in climate change knowledge to mitigate its negative effects on student health and enhance their resilience (Molthan-Hill et al., 2019; Monroe et al., 2019).

Despite this, to date, there is a paucity of studies that have explored a potential relationship between climate distress and young people’s adaptation within educational contexts themselves. Specifically, while existing evidence demonstrates an association between climate change concern and young people’s worries about their studies (e.g., Vercammen et al., 2023), research investigating whether and how these concerns may actually impair their academic experience remains lacking. This issue becomes particularly relevant when considering those students who internalize climate change as a central concern in their lives, as their distress may extend beyond emotional discomfort and interfere with core academic processes. Investigating these dynamics is essential to inform sustainable educational interventions and retention-oriented policies.

The present study aimed to investigate the relationship between climate distress, academic engagement, and burnout among university students, examining the role of academic amotivation. In detail, the growing concern about climate change consequences may be negatively related to students’ academic trajectories and wellbeing by reducing academic motivation and interest. To date, this research represents the first attempt to explore the relationship between climate distress and academic maladjustment among university students, with specific attention to academic motivational processes.

### 1.2 Climate distress, students’ wellbeing and academic maladjustment

Recent studies have shown that awareness of climate change and its consequences can lead to climate distress, characterized by a constellation of negative emotions, such as fear, guilt, and worry, which may also interfere with the cognitive sphere, resulting in difficulties with concentration and daily activities (Romano et al., 2024a; Veijonaho et al., 2025). Several studies have shown that climate distress is linked to a general decline in individuals’ wellbeing and mental health (Ogunbode et al., 2023; Prencipe et al., 2023), with young people being the most affected population (Hickman et al., 2021; Clayton and Crandon, 2024). Indeed, since the effects of climate change mitigation actions are not immediately visible, young people may perceive their efforts as ineffective, which can lead both to reduced investment in pro-environmental actions and to heightened concern and disengagement from their future life planning (e.g., Bandura and Cherry, 2020).

Future uncertainty related to climate change represents indeed a significant source of climate distress (e.g., Searle and Gow, 2010). Specifically, as documented by Jones (2023) and Vercammen et al. (2023), youths express greater concerns about their studies and careers when considering future expectations related to climate change. Furthermore, as highlighted by Diffey et al. (2022), climate distress in young people is related to impairment in performing basic tasks, such as studying or working. Climate distress has indeed been associated with increased difficulties in concentration, insomnia, and obsessive thinking, factors that may interfere with students’ performance (Pagnin et al., 2014; Clayton, 2020; Wu et al., 2020; Cosh et al., 2024).

Although there is no direct scientific evidence in this regard, it is plausible to hypothesize that in university students in the era of climate change, climate distress falls within the broader spectrum of life stress, which previous authors have shown to be predictors of academic burnout and low engagement (e.g., Huang and Lin, 2010; Lin and Huang, 2014; Maftei et al., 2024).

Academic burnout is a syndrome that arises from students’ chronic and overwhelming strain due to excessive demands. It encompasses behavioral, emotional, and cognitive aspects and is associated with severe consequences for affected students, both within and outside the academic context (i.e., academic dropout, depression, suicidal intentions, substance abuse) (Salmela-Aro and Upadyaya, 2014; Romano et al., 2019, 2020; Fiorilli et al., 2022). Academic engagement is a fulfilling state of mind characterized by vigor, dedication, and absorption in studying, and it is a relevant predictor of persistence in students’ academic tracks (Salmela-Aro and Upadyaya, 2014; Salmela-Aro, 2015).

Despite sharing features that might resemble similar but opposite phenomena, the academic literature has largely noted that burnout and engagement differ from each other and should be considered together to have a broader understanding of students’ wellbeing and adjustment (Tuominen-Soini and Salmela-Aro, 2014; Leiter and Maslach, 2017; Salmela-Aro and Read, 2017). Indeed, previous evidence has identified profiles of high-engagement and high-burnout students, corroborating different but coexisting characteristics (Salmela-Aro and Read, 2017). As shown by previous evidence, future uncertainty in environmental and life situations, as well as the sense of being out of control, have a strong negative influence on students’ burnout and engagement (Kong and Zeng, 2023; Qiang et al., 2024).

Therefore, it is possible to suppose that climate distress, as a phenomenon strongly related to future uncertainty (Searle and Gow, 2010) might be similarly related to students’ maladjustment.

### 1.3 The role of academic amotivation

Having established the relationships between climate distress and both academic burnout and engagement, it is worth investigating the psychological factors that may explain these associations. Therefore, in the present study, we propose that academic amotivation serves as a key factor in these relationships, offering a more nuanced understanding of how climate-related concerns are related to academic outcomes and reduced wellbeing.

Academic amotivation is characterized by an absence of intentionality and perceived contingency between actions and outcomes in the academic context (Vallerand et al., 1992; Ryan and Deci, 2000a). Students showing academic amotivation also show a lack of sense of direction and purpose in their educational pursuits. They experience a profound detachment from the academic environment, including disengagement from professors, coursework, and institutional activities more broadly (Legault et al., 2006; Schwan, 2021). Such students often report not understanding why they attend university or perceive their academic efforts as futile (Vallerand et al., 1992).

When students perceive environmental problems as overwhelming and beyond their control, they may experience a profound sense of helplessness that extends to their academic sphere, leading them to question whether their studies hold any meaningful purpose in the face of existential environmental threats (Hickman et al., 2021). This lack of motivation, indeed, might make students to progressively disengage from the academic context and related activities (Wigfield and Eccles, 2000; Passeggia et al., 2023). In addition, previous cross-sectional studies have shown an association between amotivated university students and academic burnout dimensions (e.g., Cabras et al., 2023).

In line with this, young people may perceive climate change as an obstacle to their educational paths and career perspectives, with increased frustration and significant repercussions on their wellbeing (Lewandowski et al., 2024). Perceptions of this kind might be reflected in academic amotivation, which within Self-Determination Theory (SDT; Ryan and Deci, 2000a,b; Vallerand et al., 1992) represents the lowest degree of self-determination along the motivational continuum, where students no longer identify any reason for engaging in their academic activities.

Furthermore, in line with the Study Demands-Resources Model (Salmela-Aro and Upadyaya, 2014), the absence of motivation and the subsequent feelings of incompetence, as well as lack of control may make students more susceptible to experiencing burnout, because they lack adequate personal resources to counteract the energy depletion process caused by continuous demands arising from the academic context.

Therefore, climate distress may be related to enhanced feelings of powerlessness and meaninglessness over one’s academic path (i.e., academic amotivation); and accordingly, it might be associated with two outcomes of poor students’ academic adjustment (i.e., burnout and low engagement).

## 2 The present study

Previous studies have shown a possible association between future concerns and negative emotionality related to climate change on the one hand, and educational and career trajectories on the other (e.g., Diffey et al., 2022). Despite this, no previous studies, as far as we know, have emerged that explore how extreme forms of climate-related distress may undermine students’ wellbeing and adjustment during their academic path, with negative repercussions in terms of reduced engagement and increased burnout. Furthermore, although climate change has been increasingly recognized as a factor that may compromise overall motivation (Stollberg and Jonas, 2021; Innocenti et al., 2023), there is a noticeable gap in the literature regarding this topic. Specifically, no substantial research has yet investigated the potential link between climate distress and academic amotivation, nor its possible role in exacerbating academic maladjustment.

Therefore, the present study aimed to investigate the extent to which climate distress may be related to either academic burnout or engagement through academic amotivation in university students. More specifically, we predicted that students showing a greater climate distress would also show a greater burnout compared to students reporting a lower climate distress (H1).

Furthermore, we proposed that climate distress would be related to academic burnout through a loss of interest and motivation toward their academic trajectory. In more details, we hypothesized that students showing a greater climate distress would also show a greater academic amotivation and, in turn, they would report a greater level of burnout (H2).

Similarly, we predicted that students showing a greater climate distress would also show lower academic engagement compared to students reporting a lower climate distress (H3). Furthermore, we expected that students’ climate distress would be related to academic engagement through students’ academic amotivation. More specifically, we hypothesized that climate distress would be related to increased academic amotivation; which in turn, would be associated with lower engagement (H4).

Furthermore, we included academic intrinsic, extrinsic, and introjected motivation as covariates in addition to the proposed model. According to Self-Determination Theory (Ryan and Deci, 2000a), motivation can be differentiated along a continuum of self-determination. Intrinsic academic motivation refers to engaging in academic activity for its inherent interest and satisfaction. Extrinsic academic motivation involves behaviors driven by external contingencies, such as rewards or social approval. Introjected academic motivation represents a partially internalized form of extrinsic motivation, in which behavior is guided by internal pressures (Vallerand et al., 1992; Zurlo et al., 2023). Previous scholars posited that different regulation types uniquely predict cognitive, affective, and behavioral outcomes (e.g., Ratelle et al., 2007; Howard et al., 2021). Therefore, controlling for autonomous and controlled regulations (e.g., intrinsic, extrinsic, introjected) helps isolate the specific contribution of amotivation to academic burnout and engagement.

**FIGURE 1:**
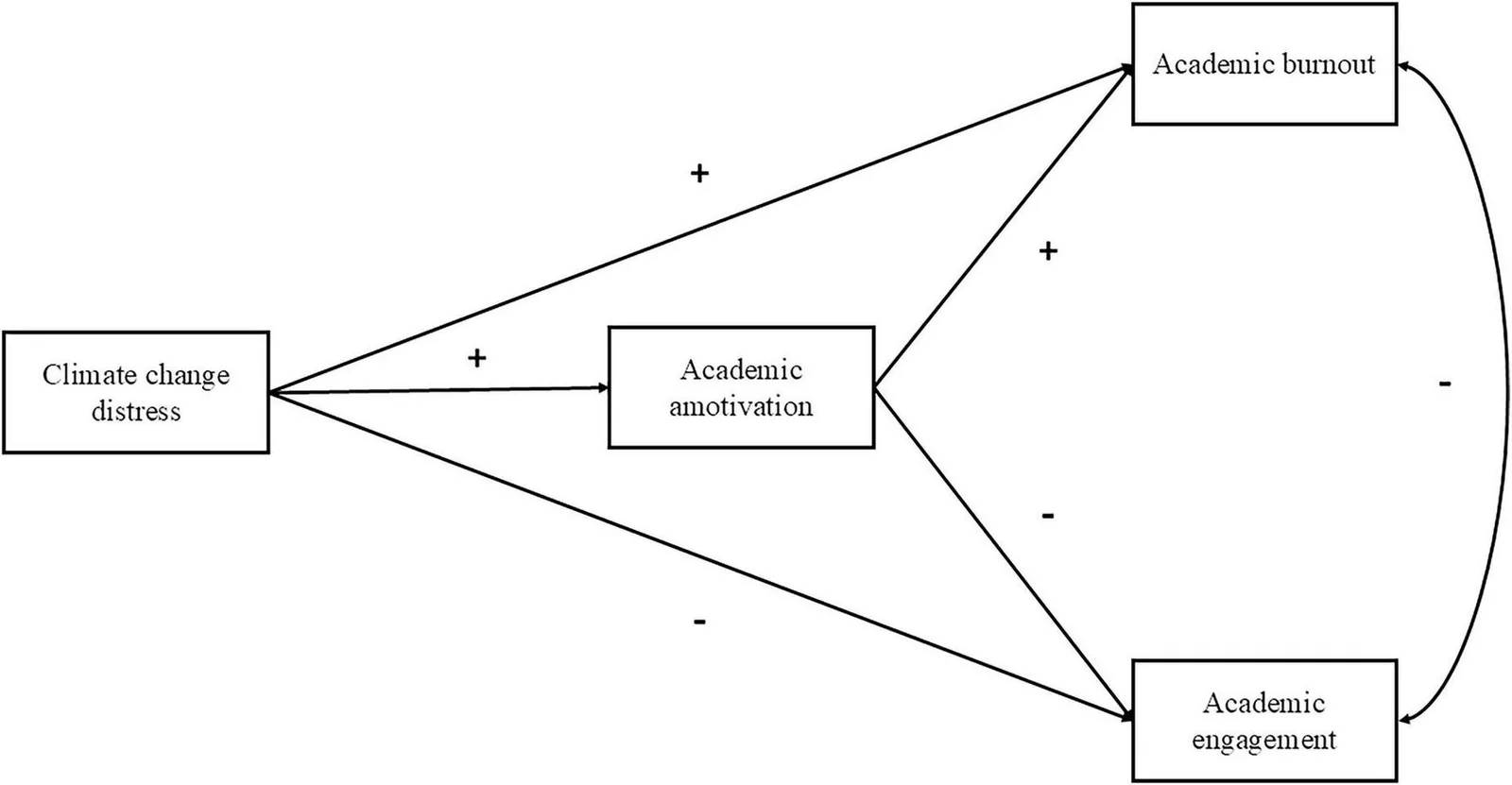
Hypothesized mediation model. Covariates (i.e., introjected motivation, intrinsic motivation, and extrinsic motivation) were not included for clarity purposes.

The hypothesized mediation model is reported in Figure 1.

## 3 Materials and methods

### 3.1 Participants and procedure

A convenience sample of 382 university students (Women = 65.4%; *M* = 21.64, SD = 2.27) was initially recruited. Data was collected between May and September 2025 using an online questionnaire. Recruitment followed a snowballing sampling strategy, with the survey being progressively shared among university students. Seven participants failed to correctly answer the attentional check (i.e., “This question is intended to assess the participant’s attention. Please select answer “4”; Likert scale from 1 to 5) and were excluded from the study.

Therefore, a final sample of 375 university students, mostly Italian (*N* = 369, 98.4%), aged from 18 to 37 years (*M* = 21.64, SD = 2.28), was included in the analyses. In detail, 244 (65.1%) participants identify themselves as women, and 131 (34.9%) identify themselves as men. Participants were both Bachelor’s degree students (*N* = 283, 75.5%) and Master’s degree students (*N* = 92, 24.5%) from both private (*N* = 225, 60%) and public universities (*N* = 150, 40%) in Italy. Concerning marital status, most of the students in the sample were single (*N* = 223, 59.5%) or in a relationship but not cohabiting (*N* = 141, 37.6%), while the remaining were cohabiting (*N* = 9, 2.4%) or married (*N* = 2, 0.5%).

Each participant completed an individual online anonymous questionnaire after providing informed consent. Participation was voluntary with no incentives offered. Anonymity was maintained throughout the survey process, with confidentiality and anonymity standards upheld at all stages of data collection. Specifically, no personal data was collected, and data were analyzed exclusively in aggregate form. Data processing followed the requirements of Article 13 of EU Regulation No. 679/2016 of 27.04.2016, the “General Data Protection Regulation” (GDPR), and Legislative Decree No. 196/2003, the “Personal Data Protection Code,” as amended by Legislative Decree No. 101 of 10.08.2018. The research protocol complies with the principles established in the Declaration of Helsinki of 1964 and its subsequent revisions. The study was approved by the Institutional Ethics Committee of the Università Europea di Roma, Rome, Italy (Prot. n. 08/2025).

### 3.2 Measures

Climate change distress: The climate change distress scale adopted by Veijonaho et al. (2024) was used to assess climate change distress. The scale was translated in Italian using a back-translation procedure. In detail, the scale encompasses eight items on a six-point Likert scale (1 = Not true at all, 6 = Completely true). An example of an item is: “Thinking about climate change makes it difficult for me to concentrate.” Cronbach’s alpha was 0.84, and McDonald’s omega was 0.87. In the present study, a total score was adopted for the climate change distress measure.

Amotivation: To assess academic amotivation, we adopted a selection of items of the Italian version of the academic motivation scale-college (AMS-C; Zurlo et al., 2023). In detail, the scale originally encompassed 26 items on a five-point Likert scale (1 = Does not correspond at all, 5 = Corresponds a lot) and includes foue subscales, namely Introjected Motivation, Intrinsic Motivation, Extrinsic Motivation, and Amotivation. The instruction was as follows: “Using the scale below, indicate to what extent each of the following items presently corresponds to one of the reasons why you go to university.” Nonetheless, to reduce participant burden and minimize the overall length of the questionnaire, we selected a subset of 12 items from the original 26-item scale, including three items per subscale. This selection, in line with previous studies adopting a similar approach (e.g., Brophy et al., 2024) was based on the items with the highest factor loadings reported in the original validation study (Zurlo et al., 2023). Furthermore, we conducted a Confirmatory Factor Analysis (CFA) on the new 12-items version of the scale using Mplus v. 8.3 (Muthén and Muthén, 2017). In detail, a Maximum Likelihood (ML) estimation with a 5,000 percentile bootstrap resample was adopted. The CFA results revealed good fit indexes: χ2 = 160.72, *p* < 0.001; RMSEA = 0.079, SRMR = 0.060, CFI = 0.95, TLI = 0.93, suggesting a correlated four-factor structure, in line with the original validation study (Zurlo et al., 2023). An example of an item of the amotivation subscale is: “I don’t know; I can’t understand what I am doing in University”; an example of an item of the introjected motivation subscale is: “Because I want to show myself that I can succeed in my studies”; an example of an item of the intrinsic motivation is: “For the “high” feeling that I experience while reading about various interesting subjects”; and an example of an item of the extrinsic motivation “In order to obtain a more prestigious job later on.” The selection of items adopted in the present study is reported in Supplementary Appendix A.

In the present study, we computed a total score across all subscales, adopting amotivation as a mediator, while the remaining three subscales were included as covariates in the model. Cronbach’s alpha and McDonald’s omega were 0.86 and 0.86 for the amotivation subscale, 0.79 and 0.79 for the introjected motivation subscale, 0.85 and 0.86 for the intrinsic motivation subscale, and 0.92 and 0.92 for the extrinsic motivation subscale, respectively.

Academic burnout: The Italian version of the Burnout Assessment Tool- Core Symptoms (BAT-C; Schaufeli et al., 2020) in its short form for university students (Fiorilli et al., 2022) was used to assess academic burnout. The scale encompasses 12 items on a five-point Likert scale (1 = Never, 5 = Always). An example of an item is: “Due to my studies, I feel mentally exhausted.” Cronbach’s alpha and McDonald’s omega were 0.86 and 0.87, respectively. In the present study, a total score was adopted for the academic burnout measure.

Academic engagement: The Utrecht Work Engagement Scale 9 Items (Schaufeli et al., 2006), in its Italian validation for students (UWES-S-9; Loscalzo and Giannini, 2019) was used to assess academic engagement. The scale encompasses nine items on a seven-point Likert scale (0 = Never, 6 = Always). An example of an item is: “When I study, I feel full of energy.” Cronbach’s alpha and McDonald’s omega were 0.92 and 0.92, respectively. In the present study, a total score was adopted for the academic engagement measure.

## 4 Results

To evaluate whether the sample size was adequate to detect the hypothesized indirect associations, we conducted a Monte Carlo power analysis for indirect effects (Schoemann et al., 2017). As the present associations had not been previously examined, and in line with recommendations on typical effect sizes in psychological research (Gignac and Szodorai, 2016), we set the correlations among the independent variable, the mediator, and the dependent variable to *r* = 0.20 (i.e., a medium effect size). The analysis was based on 5,000 replications with 20,000 Monte Carlo draws per replication (random seed = 1,234) and a target power of 0.80. Results indicated that the achieved sample size (*N* = 375) provided a power of approximately 0.87 to detect the indirect effect.

A path analysis with Mplus v. 8.3 (Muthén and Muthén, 2017) was performed to verify the hypothesized mediation model. In detail, climate change distress was the independent variable, amotivation the mediator, and academic burnout and engagement the dependent variables. All variables were treated as observed variables, each composed of the sum of the respective items. Moreover, academic introjected motivation, academic intrinsic motivation, and academic extrinsic motivation were included as covariates in the model. Maximum Likelihood (ML) estimation with 5,000 percentile bootstrap resampling was used, and the MODEL INDIRECT command was adopted to obtain the desired indirect associations. To assess the model’s goodness of fit, the following indices were considered: the chi-squared test (a *p*-value greater than 0.05 indicates good fit); the Comparative Fit Index (CFI) and the Tucker-Lewis Index (TLI), with values of 0.90 or higher indicating good fit and values of 0.95 or higher indicating excellent fit; and the Root Mean Square Error of Approximation (RMSEA) and the Standardized Root Mean Square Residual (SRMR), for which values of 0.08 or lower suggest good fit, and values of 0.05 or lower suggest very good fit. As often occurs in path analysis, such a model is just-identified (i.e., saturated), with zero degrees of freedom (df = 0); in just-identified models, the observed covariance matrix is reproduced exactly, so fit indices are perfect by construction (e.g., χ^2^ = 0, CFI = 1.00) and reflect the saturated specification rather than goodness of fit (Kline, 2023; MacCallum and Austin, 2000). Accordingly, and consistent with recent methodological recommendations (Stone, 2021), inference was based on the statistical significance and substantive coherence of the individual parameter estimates rather than on global fit indices.

**FIGURE 2:**
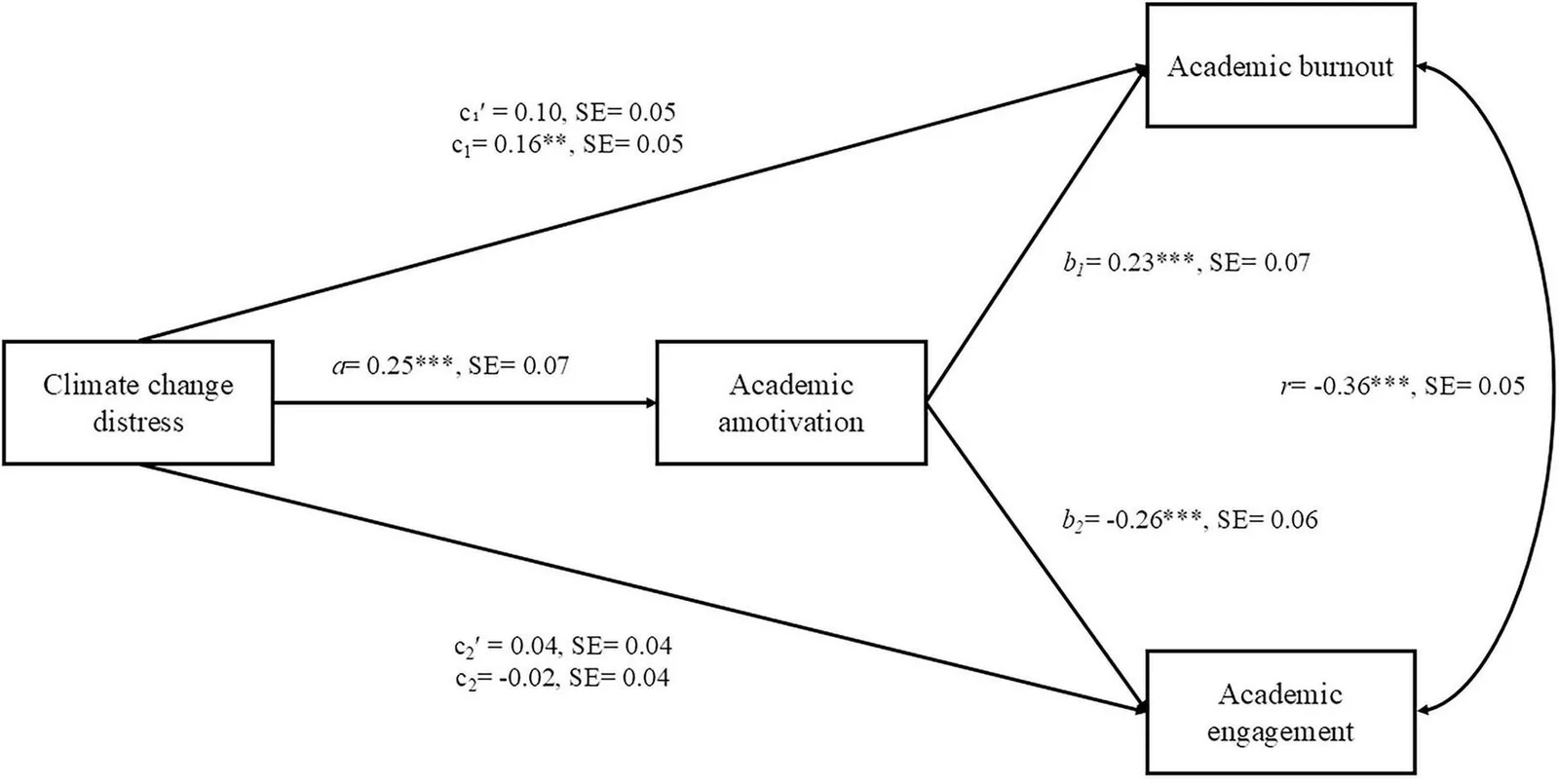
Mediation model results. **p < 0.01, ***p < 0.001. Results are standardized. The covariate associations were not included for clarity purposes; c_1_’, direct path climate change distress-academic burnout; c_1_, total association of the path climate change distress-academic burnout; c_2_’, direct path climate change distress-academic engagement; c_2_, total association of the path climate change distress-academic engagement.

Mediation model results are reported in Figure 2.

As the tested model was just-identified (df = 0), it reproduced the observed covariance matrix exactly, yielding perfect fit values by construction (χ^2^ = 0.00; RMSEA = 0.00; SRMR = 0.00; CFI = 1.00; TLI = 1.00). Results revealed that climate change distress was significantly and positively related to academic amotivation (see path a, Figure 2). Moreover, academic amotivation was significantly and positively related to academic burnout (see path b_1_, Figure 2) and significantly and negatively related to academic engagement (see path b_2_, Figure 2). A significant negative relationship emerges between academic burnout and engagement (see Figure 2). Concerning covariates, both introjected (β = 0.11, SE = 0.06, 95% BootCI [0.01 to 0.20]) and extrinsic academic motivation (β = 0.10, SE = 0.05, 95% BootCI [0.02 to 0.19]) were positively related to academic burnout. By contrast, intrinsic academic motivation was negatively related to academic burnout (β = −0.19, SE = 0.04, 95% BootCI [−0.28 to −0.09]). Moreover, only intrinsic academic motivation among the covariates was significantly and positively related to academic engagement (β = 0.42, SE = 0.05, 95% BootCI [0.34 to 0.50]). Furthermore, only intrinsic motivation among the covariates was significantly and negatively related to academic amotivation (β = −0.19, SE = 0.05, 95% BootCI [−0.27 to −0.11]). Results revealed a significant indirect association between climate change distress and academic burnout through academic amotivation (standardized point estimate = 0.06, SE = 0.02, 95% BootCI [0.02 to 0.09]). The direct association was non-significant (see path c_1_’ in Figure 2) while the total association was significant (see path c_1_ in Figure 2). Finally, results revealed a significant negative indirect association between climate change distress and academic engagement through academic amotivation (standardized point estimate = −0.06, SE = 0.02, 95% BootCI [−0.10 to −0.03]). The total and direct associations were both non-significant (see path c_2_’ and c_2_ in Figure 2). Nonetheless, according to Hayes (2009) and other scholars (e.g., MacKinnon et al., 2000), researchers do not need to establish a significant total association before examining indirect associations. As highlighted by Hayes (2009, pp. 414–415), the lack of deepening indirect effects in the absence of a total effect may result in overlooking meaningful, relevant, or informative mechanisms through which X may influence Y.

## 5 Discussion and conclusion

### 5.1 Discussion

The present study aimed to investigate, in a convenience sample of university students, the associations between climate distress on the one hand and academic engagement as well as burnout, on the other hand; hypothesizing that climate distress may be related to both engagement and burnout through academic amotivation. Specifically, it was hypothesized that climate distress would be associated with greater academic burnout (H1) and lower academic engagement (H3). Furthermore, it was further hypothesized that students’ climate distress would be linked to such outcomes (i.e., academic engagement and burnout) through academic amotivation. In detail, theoretically climate distress would be associated with increased academic amotivation, which in turn, would be related to higher academic burnout (H2) and lower engagement (H4).

Our findings, together with theoretical propositions, revealed an indirect association between climate distress and both academic burnout and engagement, through academic amotivation. Nonetheless no direct associations emerged between climate distress and burnout and between climate distress and engagement. Notably, the association noted was specific to amotivation and emerged over and above intrinsic, extrinsic, and introjected motivation, which were controlled for. This suggests that what relates climate distress to lower engagement and higher burnout is not the quality of students’ motivation, but rather the perception that engaging in academic activities is not worthwhile or does not lead to valued outcomes. However, because we only have cross-sectional data, these propositions about direction of influence should be interpreted with caution. Accordingly, the interpretations below are as theoretically plausible readings of the observed pattern rather than as causal mechanisms.

Indeed, reading our findings through the lens of the Self-Determination Theory (SDT; Ryan and Deci, 2000a,b, 2024), it is tempting to speculate that climate distress may be accompanied by lower students’ perceptions of competence and autonomy, contributing to a greater sense of losing control over their behavior. Given the uncertainty surrounding future prospects and the negative emotional experiences associated with climate change concerns (Pihkala, 2020; Jones, 2023; Vercammen et al., 2023), students may experience heightened perceptions of helplessness, which in turn, might be linked to the sense of purpose they ascribe to both their academic pathway and prospective career development. Previous research has indeed identified that the perception of uncontrollability of climate change is a constitutive element of climate distress (e.g., Searle and Gow, 2010), and it is possible to speculate that this perceived lack of control may also be reflected in students’ sense of control over their academic path and trajectories, and might co-occur with their lack of motivation. The perception of uncontrollability, reflecting thwarted or unsatisfied basic psychological needs, might co-occur with increased meaninglessness and frustration, as well as with reduced engagement and heightened burnout (Bartholomew et al., 2011a,b; Costa et al., 2015). Nonetheless, it is worth noting that, although we measured the motivational components of the SDT, we did not include specific measures of basic psychological needs (i.e., autonomy, competence and relatedness), nor measures on perception of controllability in the current study. Therefore, the interpretations above should be taken with caution and verified in future studies, as they represents theoretical interpretations rather that findings supported by the data.

Furthermore, drawing on the Conservation of Resources Theory (COR; Hobfoll, 2011), it is tempting to speculate that climate distress may represent a chronic stressor associated with the depletion of psychological resources in students’ everyday lives. In this vein, broadly speaking, it could be possible that academic amotivation may act as a dysfunctional adaptive mechanism in order to preserve the available resources. As a result of an ineffective strategy, it might be associated with exhaustion and lower academic engagement. Indeed, previous research has consistently shown that academic amotivation is negatively associated with key academic outcomes, including lower engagement, reduced achievement, and even more severe forms of academic maladjustment (e.g., Balkis, 2018; Felaza et al., 2020; Howard et al., 2021; Ilter, 2021). Nonetheless, longitudinal designs would be needed to test whether such a process unfolds over time (Alarcon et al., 2011).

A further aspect worth considering is the absence of direct associations between climate distress and either engagement or burnout. Engagement and burnout are outcomes that emerge from processes embedded within the academic context, whereas climate distress reflects a broader, distal concern that is not rooted in that domain. It is therefore plausible that climate distress relates to these academic outcomes not directly, but only in conjunction with a proximal, domain-specific correlate (i.e., academic amotivation), consistent with our mediation findings. One tentative reading of why this distal concern should be associated specifically with academic motivation relates to the future-oriented nature of studying, which is inherently an investment in future goals and prospects: climate distress and anxiety have been linked to a more threatened view of the future and to a weakened sense of future self-continuity (Hickman et al., 2021; Qin et al., 2024), a construct we did not assess here. This interpretation therefore remains speculative and, together with the cross-sectional design, calls for confirmation in future research.

Moreover, the inverse relationship between burnout and engagement observed in the present study is consistent with findings extensively documented in previous research (e.g., Schaufeli and Buunk, 2002; Cazan, 2015). The magnitude of this relationship in our data further corroborates that these constructs, while related, are conceptually distinct (Salmela-Aro and Read, 2017). Regarding covariates, the observed associations align with prior empirical evidence. Specifically, previous studies have demonstrated that controlled motivation, encompassing both introjected and external regulation, tends to be positively associated with students’ burnout (e.g., Chang et al., 2016; Lee, 2024). Conversely, in line with previous findings (e.g., Chang et al., 2016; Güngör and Sari, 2022; Cho et al., 2023), intrinsic motivation appears to function as a protective factor against academic maladjustment, being inversely associated with academic burnout and positively related to academic engagement. Finally, results concerning the relationship between intrinsic motivation and amotivation align with the Self-Determination Theory assumptions (Ryan and Deci, 2000b).

Taken together, our findings highlight that climate distress may represent a stressor for contemporary and future generations of university students, which is associated with their academic pursuits. More specifically, despite warranting further investigations, the results of the current study suggest that climate distress is negatively associated with students’ sense of pursuit over their academic path (i.e., academic amotivation), which is linked to lower positive engagement with their studies and higher forms of academic maladjustment, such as burnout.

### 5.2 Strengths, limitations, and future directions

To the best of our knowledge, this is the first study to examine the relationship between climate distress and academic maladjustment among university students, as well as the first study to explore these relationships within a unified model. Moreover, no previous studies have presented a motivational process that may help to explain how climate distress is related to academic burnout and reduced engagement among students.

Despite the highlighted strengths, our study has several limitations that should be acknowledged. First, the study employed a cross-sectional design, which does not allow for establishing causal relationships among the variables under investigation. It could, for instance, be that it is burnout that influences amotivation rather than that amotivation influences burnout, or most probably, the direction of influence could be bidirectional. Future research adopting a longitudinal design should further examine the relationships among the studied variables over time, with additional attention to contextual factors such as faculty and peer support, aspects not considered in the present study.

Another limitation concerns the omission of potentially relevant psychological and contextual variables, such as general anxiety, depression, academic stress, psychological wellbeing, and social or family support. Therefore, we cannot exclude the possibility that climate distress may partially reflect broader psychological distress rather than acting as a unique specific predictor of academic maladjustment. Although the adopted measure specifically assessed climate-related distress, future studies should examine the incremental contribution of climate distress over and above general distress-related constructs to better clarify its role in students’ academic functioning.

A further limitation concerns the magnitude of the observed effects. Although the indirect effects of climate distress on burnout (β = 0.06) and engagement (β = −0.06) reached statistical significance, and the bivariate associations were small to moderate, effects of this size are estimated with limited precision in a sample of 375 participants. The statistical significance of such small effects should therefore not be equated with strong practical relevance, and climate distress is best understood as a modest correlate of students’ academic functioning. Accordingly, our findings and related implications should be interpreted with caution and confirmed in larger samples that allow small indirect effects to be estimated more precisely.

Furthermore, although the sample includes both public and private universities, it does not allow generalization of the findings to the entire student population. Future studies could replicate these results with students from international universities, thereby further consolidating the observed results. In addition, it would be valuable to explore these findings by differentiating a priori the student population from non-Western countries, which are typically underinvestigated.

Finally, a further limitation concerns the use of the 12-item short form of the AMS-C. Although the items were selected on the basis of the factor loadings reported in the original validation study and the four-factor structure was confirmed with an adequate fit and good internal consistency in the present sample, the shortened version was examined using the same sample that was subsequently used to test the substantive hypotheses. Consequently, it was not cross-validated using an independent sample. This restricts the strength of the psychometric evidence for the instrument and prevents us from fully ruling out sample-specific influences. Future research should validate this short form in independent samples, ideally through a dedicated psychometric study, before it is adopted more widely.

#### 5.3 Implications for future practice

Given the indirect nature and the small magnitude of the associations outlined in the present study, the following considerations are offered as directions for future research and as questions that the design and evaluation of educational initiatives might address.

A first question concerns structured climate change education programs that explicitly acknowledge the psycho-physical strain associated with more intense forms of climate distress: future studies could examine whether participation in such programs is associated with more adaptive academic outcomes. Existing work has discussed early opportunities to recognize and elaborate climate-related distress as potentially valuable, together with initiatives addressing students’ sense of agency and resilience through the collaborative discussion of functional coping strategies (e.g., Leichenko and O’Brien, 2020). Whether these initiatives are related to the motivational pathway examined in the present study remains to be tested.

Such research seems warranted given evidence that young people, who are among those most exposed to the consequences of climate change and who represent future stakeholders and policymakers, remain marginal in climate-related decision-making processes (O’Brien et al., 2018; Romano et al., 2024b). Longitudinal and, ideally, intervention designs would be required to establish whether addressing climate change within educational settings is associated with a stronger sense of connection, belonging, and engagement among students, and with more sustained academic motivation under conditions of future uncertainty (e.g., Allen et al., 2024).

More broadly, the present results are consistent with the idea that climate change may represent not only an environmental issue but also a psychological and educational one, in that climate distress appeared to be associated, with academic amotivation and, through it, with engagement and maladjustment. Should this pattern be replicated with stronger designs, it would suggest that education may function not only as preparation for the future but also as a context in which the emotional and motivational resources for facing climate change are developed.

### 5.4 Conclusion

The present study aimed to investigate the relationship between climate distress and both academic engagement and burnout in a sample of university students. Furthermore, the key role of academic amotivation in these relationships was examined. Results partially confirmed the hypotheses formulated: climate distress is indirectly related to higher burnout and lower engagement through academic amotivation. Nonetheless, no direct associations between climate distress, academic burnout and academic engagement were detected. This study represents the first attempt to investigate, within a unified model, the relationship between climate distress and academic maladjustment by examining the motivational dynamics involved. These findings point to academic amotivation as a candidate focus for future work on how climate distress relates to students’ functioning in higher education. Whether initiatives addressing students’ sense of agency in relation to climate change are associated with more favorable psycho-physical wellbeing and academic trajectories is a question that longitudinal and intervention designs will need to answer.

## Data Availability

The raw data supporting the conclusions of this article will be made available by the authors, without undue reservation.

## References

[B1] Abbass K. Qasim M. Z. Song H. Murshed M. Mahmood H. Younis I. (2022). A review of the global climate change impacts, adaptation, and sustainable mitigation measures. *Environ. Sci. Pollut. Res. Int*. 29 42539–42559. 10.1007/s11356-022-19718-6 35378646 PMC8978769

[B2] Alarcon G. M. Edwards J. M. Menke L. E. (2011). Student burnout and engagement: A test of the conservation of resources theory. *J. Psychol*. 145 211–227. 10.1080/00223980.2011.555432 21560805

[B3] Allen K.-A. Slaten C. Lan M. Craig H. May F. Counted V. (2024). Belonging in higher education: A twenty-year systematic review. *J. Univ. Teach. Learn. Pract.* 21 1–55. 10.53761/s2he6n66

[B4] Balkis M. (2018). Academic amotivation and intention to school dropout: The mediation role of academic achievement and absenteeism. *Asia Pac. J. Educ.* 38 257–270. 10.1080/02188791.2018.1460258

[B5] Bandura A. Cherry L. (2020). Enlisting the power of youth for climate change. *Am. Psychol*. 75 945–951. 10.1037/amp0000512 31436438

[B6] Bartholomew K. J. Ntoumanis N. Ryan R. M. Thøgersen-Ntoumani C. (2011b). Psychological need thwarting in the sport context: Assessing the darker side of athletic experience. *J. Sport. Exerc. Psychol*. 33 75–102. 10.1123/jsep.33.1.75 21451172

[B7] Bartholomew K. J. Ntoumanis N. Ryan R. M. Bosch J. A. Thøgersen-Ntoumani C. (2011a). Self-determination theory and diminished functioning: The role of interpersonal control and psychological need thwarting. *Pers. Soc. Psychol. Bull*. 37 1459–1473. 10.1177/0146167211413125 21700794

[B8] Brophy K. Emery M. Coroiu A. Albani C. Braehler E. Körner A. (2024). Development and validation of a short form of the German self-consciousness scale for the general population. *Eur. J. Psychol. Open* 83 173–185. 10.1024/2673-8627/a000068

[B9] Cabras C. Konyukhova T. Lukianova N. Mondo M. Sechi C. (2023). Gender and country differences in academic motivation, coping strategies, and academic burnout in a sample of Italian and Russian first-year university students. *Heliyon* 9:e16617. 10.1016/j.heliyon.2023.e16617 37260901 PMC10227335

[B10] Cazan A.-M. (2015). Learning motivation, engagement and burnout among university Students. *Procedia. Soc. Behav. Sci.* 187 413–417. 10.1016/j.sbspro.2015.03.077

[B11] Chang E. Lee A. Byeon E. Seong H. Lee S. M. (2016). The mediating effect of motivational types in the relationship between perfectionism and academic burnout. *Pers. Individ. Dif.* 89 202–210. 10.1016/j.paid.2015.10.010

[B12] Cho S. Lee M. Lee S. M. (2023). Burned-out classroom climate, intrinsic motivation, and academic engagement: Exploring unresolved issues in the job demand-resource model. *Psychol. Rep*. 126 1954–1976. 10.1177/00332941211054776 35212248

[B13] Cianconi P. Betrò S. Janiri L. (2020). The impact of climate change on mental health: A systematic descriptive review. *Front. Psychiatry*. 11:74. 10.3389/fpsyt.2020.00074 32210846 PMC7068211

[B14] Clayton S. (2020). Climate anxiety: Psychological responses to climate change. *J. Anxiety. Disord*. 74:102263. 10.1016/j.janxdis.2020.102263 32623280

[B15] Clayton S. Crandon T. (2024). “Climate distress among young people,” in *Climate Change and Youth Mental Health: Multidisciplinary Perspectives*, eds Haase E. Hudson K. (Cambridge: Cambridge University Press).

[B16] Clemens V. von Hirschhausen E. Fegert J. M. (2022). Report of the intergovernmental panel on climate change: Implications for the mental health policy of children and adolescents in Europe-a scoping review. *Eur. Child Adolesc. Psychiatry*. 31 701–713. 10.1007/s00787-020-01615-3 32845381 PMC9142437

[B17] Cosh S. M. Ryan R. Fallander K. Robinson K. Tognela J. Tully P. J. et al. (2024). The relationship between climate change and mental health: A systematic review of the association between eco-anxiety, psychological distress, and symptoms of major affective disorders. *BMC Psychiatry* 24:833. 10.1186/s12888-024-06274-1 39567913 PMC11577747

[B18] Costa S. Ntoumanis N. Bartholomew K. J. (2015). Predicting the brighter and darker sides of interpersonal relationships: Does psychological need thwarting matter? *Motiv. Emot.* 39 11–24. 10.1007/s11031-014-9427-0

[B19] Diffey J. Wright S. Uchendu J. O. Masithi S. Olude A. Juma D. O. et al. (2022). “Not about us without us” - the feelings and hopes of climate-concerned young people around the world. *Int. Rev. Psychiatry* 34 499–509. 10.1080/09540261.2022.2126297 36165749

[B20] Felaza E. Findyartini A. Setyorini D. Mustika R. (2020). How motivation correlates with academic burnout: Study conducted in undergraduate medical students. *Educ. Med. J* 12 43–52. 10.21315/eimj2020.12.1.5 PMC7346292 32685671

[B21] Fiorilli C. Barni D. Russo C. Marchetti V. Angelini G. Romano L. (2022). Students’ burnout at university: The role of gender and worker status. *Int. J. Environ. Res. Public Health* 19:11341. 10.3390/ijerph191811341 36141612 PMC9517191

[B22] Gignac G. E. Szodorai E. T. (2016). Effect size guidelines for individual differences researchers. *Pers. Individ. Dif.* 102 74–78. 10.1016/j.paid.2016.06.069

[B23] Gislason M. K. Kennedy A. M. Witham S. M. (2021). The interplay between social and ecological determinants of mental health for children and youth in the climate crisis. *Int. J. Environ. Res. Public Health* 18:4573. 10.3390/ijerph18094573 33925907 PMC8123462

[B24] Güngör A. Sari H. I. (2022). Effects of academic motivation on school burnout in Turkish college students. *Int. J. Adv. Couns*. 44 414–431. 10.1007/s10447-022-09477-x 35791378 PMC9247920

[B25] Hayes A. F. (2009). Beyond Baron and Kenny: Statistical mediation analysis in the new millennium. *Commun. Monogr.* 76 408–420. 10.1080/03637750903310360

[B26] Hickman C. Marks E. Pihkala P. Clayton S. Lewandowski R. E. Mayall E. E. et al. (2021). Climate anxiety in children and young people and their beliefs about government responses to climate change: A global survey. *Lancet Planet. Health* 5 e863–e873. 10.1016/S2542-5196(21)00278-3 34895496

[B27] Hobfoll S. E. (2011). “Conservation of resources theory: Its implication for stress, health, and resilience,” in *The Oxford Handbook of Stress, Health, and Coping*, ed. Folkman S. (Oxford, NY: Oxford University Press), 127–147.

[B28] Howard J. L. Bureau J. Guay F. Chong J. X. Y. Ryan R. M. (2021). Student motivation and associated outcomes: A meta-analysis from self-determination theory. *Perspect. Psychol. Sci*. 16 1300–1323. 10.1177/1745691620966789 33593153

[B29] Huang Y.-C. Lin S. H. (2010). Canonical correlation analysis on life stress and learning burnout of college students in Taiwan. *Int. Electron. J. Health Educ* 13 145–155.

[B30] Hwong A. R. Wang M. Khan H. Chagwedera D. N. Grzenda A. Doty B. et al. (2022). Climate change and mental health research methods, gaps, and priorities: A scoping review. *Lancet Planet. Health* 6 e281–e291. 10.1016/S2542-5196(22)00012-2 35278392

[B31] Ilter İ (2021). The relationship between academic amotivation and academic achievement: A study on middle school students. *J. Theor. Educ. Sci* 14 389–410. 10.30831/akukeg.847145

[B32] Innocenti M. Santarelli G. Lombardi G. S. Ciabini L. Zjalic D. Di Russo M. et al. (2023). How can climate change anxiety induce both pro-environmental behaviours and eco-paralysis? The mediating role of general self-efficacy. *Int. J. Environ. Res. Public Health* 20:3085. 10.3390/ijerph20043085 36833780 PMC9960236

[B33] IPCC (2023). “Climate change 2023: Synthesis report, summary for policymakers,” in *Contribution of Working Groups I, II and III to the Sixth Assessment Report of the Intergovernmental Panel on Climate Change*, eds Lee H. Romero J. (Geneva: IPCC). 10.1111/gcb.16585

[B34] Jones C. A. (2023). Life in the shadows: Young people’s experiences of climate change futures. *Futures* 154:103264. 10.1016/j.futures.2023.103264

[B35] Kline R. B. (2023). *Principles and Practice of Structural Equation Modeling.* New York, NY: Guilford publications.

[B36] Kong T. Zeng S. (2023). The effect of perceived environmental uncertainty on university students’ anxiety, academic engagement, and prosocial behavior. *Behav. Sci.* 13:906. 10.3390/bs13110906 37998653 PMC10669797

[B37] Lee M. (2024). Introjected regulation and academic burnout: A moderated mediation model of social comparison and distress overtolerance. *Stress Health* 40:e3381. 10.1002/smi.3381 38380890

[B38] Legault L. Green-Demers I. Pelletier L. (2006). Why do high school students lack motivation in the classroom? Toward an understanding of academic amotivation and the role of social support. *J. Educ. Psychol.* 98:567. 10.1037/0022-0663.98.3.567

[B39] Leichenko R. O’Brien K. (2020). Teaching climate change in the anthropocene: An integrative approach. *Anthropocene* 30:100241. 10.1016/j.ancene.2020.100241

[B40] Leiter M. P. Maslach C. (2017). Burnout and engagement: Contributions to a new vision. *Burn. Res.* 5 55–57. 10.1016/j.burn.2017.04.003

[B41] Lewandowski R. E. Clayton S. D. Olbrich L. Sakshaug J. W. Wray B. Schwartz S. E. O. et al. (2024). Climate emotions, thoughts, and plans among US adolescents and young adults: A cross-sectional descriptive survey and analysis by political party identification and self-reported exposure to severe weather events. *Lancet Planet. Health* 8 e879–e893. 10.1016/S2542-5196(24)00229-8 39427673

[B42] Lin S.-H. Huang Y.-C. (2014). Life stress and academic burnout. *Act. Learn. High. Educ.* 15 77–90. 10.1177/1469787413514651

[B43] Loscalzo Y. Giannini M. (2019). Study engagement in Italian university students: A confirmatory factor analysis of the Utrecht work engagement scale—student version. *Soc. Indic. Res.* 142 845–854. 10.1007/s11205-018-1943-y

[B44] MacCallum R. C. Austin J. T. (2000). Applications of structural equation modeling in psychological research. *Annu. Rev. Psychol*. 51 201–226. 10.1146/annurev.psych.51.1.201 10751970

[B45] MacKinnon D. P. Krull J. L. Lockwood C. M. (2000). Equivalence of the mediation, confounding and suppression effect. *Prev. Sci*. 1 173–181. 10.1023/a:1026595011371 11523746 PMC2819361

[B46] Maftei A. Opariuc-Dan C. Vrabie T. (2024). Moral disengagement and academic engagement: The moderating roles of educational anti-mattering and psychological distress. *Ethics Behav.* 34 342–359. 10.1080/10508422.2023.2225203

[B47] Manning C. Clayton S. (2018). “Threats to mental health and wellbeing associated with climate change,” in *Psychology and Climate Change: Human perceptions, impacts, and responses*, eds Clayton S. Manning C.. (London: Elsevier Academic Press), 217–244. 10.1016/B978-0-12-813130-5.00009-6

[B48] Molthan-Hill P. Worsfold N. Nagy G. J. Leal Filho W. Mifsud M. (2019). Climate change education for universities: A conceptual framework from an international study. *J. Clean. Prod.* 226 1092–1101. 10.1016/j.jclepro.2019.04.053

[B49] Monroe M. C. Plate R. R. Oxarart A. Bowers A. Chaves W. A. (2019). Identifying effective climate change education strategies: A systematic review of the research. *Environ. Educ. Res.* 25 791–812. 10.1080/13504622.2017.1360842

[B50] Muthén B. Muthén L. (2017). “Mplus,” in *Handbook of Item Response Theory*, ed. van der Linden W. J.. (Boca Raton, FL: Chapman and Hall/CRC), 507–518.

[B51] O’Brien K. Selboe E. Hayward B. M. (2018). Exploring youth activism on climate change: Dutiful, disruptive, and dangerous dissent. *Ecol. Soc.* 23:art42. 10.5751/ES-10287-230342

[B52] Ogunbode C. A. Pallesen S. Böhm G. Doran R. Bhullar N. Aquino S. et al. (2023). Negative emotions about climate change are related to insomnia symptoms and mental health: Cross-sectional evidence from 25 countries. *Curr. Psychol.* 42 845–854. 10.1007/s12144-021-01385-4

[B53] Pagnin D. de Queiroz V. Carvalho Y. T. Dutra A. S. Amaral M. B. Queiroz T. T. (2014). The relation between burnout and sleep disorders in medical students. *Acad. Psychiatry* 38 438–444. 10.1007/s40596-014-0093-z 24683060

[B54] Passeggia R. Testa I. Esposito G. Picione R. D. L. Ragozini G. Freda M. F. (2023). Examining the relation between first-year university students’ intention to drop-out and academic engagement: The role of motivation, subjective well-being and retrospective judgements of school experience. *Innov. High. Educ.* 48 837–859. 10.1007/s10755-023-09674-5

[B55] Pihkala P. (2020). Eco-anxiety and environmental education. *Sustainability* 12:10149. 10.3390/su122310149

[B56] Prencipe L. Houweling T. A. J. van Lenthe F. J. Kajula L. Palermo T. (2023). Climate distress, climate-sensitive risk factors, and mental health among Tanzanian youth: A cross-sectional study. *Lancet Planet. Health*. 7 e877–e887. 10.1016/S2542-5196(23)00234-6 37940208

[B57] Qiang J. He X. Xia Z. Huang J. Xu C. (2024). The association between intolerance of uncertainty and academic burnout among university students: The role of self-regulatory fatigue and self-compassion. *Front. Public Health* 12:1441465. 10.3389/fpubh.2024.1441465 39114523 PMC11303341

[B58] Qin Z. Wu Q. Bi C. Deng Y. Hu Q. (2024). The relationship between climate change anxiety and pro-environmental behavior in adolescents: The mediating role of future self-continuity and the moderating role of green self-efficacy. *BMC Psychol*. 12:241. 10.1186/s40359-024-01746-1 38678287 PMC11056057

[B59] Ratelle C. F. Guay F. Vallerand R. J. Larose S. Senécal C. (2007). Autonomous, controlled, and amotivated types of academic motivation: A person-oriented analysis. *J. Educ. Psychol.* 99:734. 10.1037/0022-0663.99.4.734

[B60] Rocque R. J. Beaudoin C. Ndjaboue R. Cameron L. Poirier-Bergeron L. Poulin-Rheault R. A. et al. (2021). Health effects of climate change: An overview of systematic reviews. *BMJ Open* 11:e046333. 10.1136/bmjopen-2020-046333 34108165 PMC8191619

[B61] Romano L. Buonomo I. Callea A. Fiorilli C. (2019). Alexithymia in young people’s academic career: The mediating role of anxiety and resilience. *J. Genet. Psychol*. 180 157–169. 10.1080/00221325.2019.1620675 31165680

[B62] Romano L. Buonomo I. Callea A. Fiorilli C. Schenke K. (2020). Teacher emotional support scale on Italian high school students: A contribution to validation. *Open Psychol. J.* 13 123–132. 10.2174/1874350102013010123

[B63] Romano L. Russo C. Carbone G. A. Clemente D. Imperatori C. Fiorilli C. et al. (2024a). Adolescents’ climate anxiety is related to participation in pro-environmental movements through social media usage: Boys show greater associations than girls. *Ecopsychology* 16 158–171. 10.1089/eco.2023.0013

[B64] Romano L. Russo C. Gladwin T. E. Panno A. (2024b). Adolescents and young adults’ participation in pro-environmental movements: A systematic review. *J. Genet. Psychol.* 185, 373–398. 10.1080/00221325.2024.2316804 38373092

[B65] Ryan R. M. Deci E. L. (2000a). Intrinsic and extrinsic motivations: Classic definitions and new directions. *Contemp. Educ. Psychol*. 25 54–67. 10.1006/ceps.1999.1020 10620381

[B66] Ryan R. M. Deci E. L. (2000b). Self-determination theory and the facilitation of intrinsic motivation, social development, and well-being. *Am. Psychol*. 55 68–78. 10.1037//0003-066x.55.1.68 11392867

[B67] Ryan R. M. Deci E. L. (2024). “Self-determination theory,” in *Encyclopedia of quality of life and well-being research*, ed. Maggino F. (Cham: Springer), 6229–6235.

[B68] Salmela-Aro K. (2015). Toward a new science of academic engagement. *Res. Hum. Dev.* 12 304–311. 10.1080/15427609.2015.1068038

[B69] Salmela-Aro K. Read S. (2017). Study engagement and burnout profiles among finnish higher education students. *Burn. Res.* 7 21–28. 10.1016/j.burn.2017.11.001

[B70] Salmela-Aro K. Upadyaya K. (2014). School burnout and engagement in the context of demands-resources model. *Br. J. Educ. Psychol*. 84 137–151. 10.1111/bjep.12018 24547758

[B71] Schaufeli W. B. Buunk B. P. (2002). “Burnout: An overview of 25 years of research and theorizing,” in *The Handbook of Work and Health Psychology*, eds Schabracq M. J. Winnubst A. M. Cooper C. L. (Chichester: Wiley), 383–425. 10.1002/0470013400.ch19

[B72] Schaufeli W. B. Bakker A. B. Salanova M. (2006). The measurement of work engagement with a short questionnaire: A cross-national study. *Educ. Psychol. Meas.* 66 701–716. 10.1177/0013164405282471

[B73] Schaufeli W. B. Desart S. De Witte H. (2020). Burnout Assessment Tool (BAT)-Development, Validity, and Reliability. *Int. J. Environ. Res. Public Health* 17:9495. 10.3390/ijerph17249495 33352940 PMC7766078

[B74] Schoemann A. M. Boulton A. J. Short S. D. (2017). Determining power and sample size for simple and complex mediation models. *Soc. Psychol. Personal. Sci.* 8 379–386. 10.1177/1948550617715068

[B75] Schwan A. (2021). Perceptions of student motivation and amotivation. *Clearing House J. Educ. Strategie, Issues Ideas* 94 76–82. 10.1080/00098655.2020.1867490

[B76] Searle K. Gow K. (2010). Do concerns about climate change lead to distress? *Int. J. Clim. Chang. Strateg. Manag.* 2 362–379. 10.1108/17568691011089891

[B77] Stollberg J. Jonas E. (2021). Existential threat as a challenge for individual and collective engagement: Climate change and the motivation to act. *Curr. Opin. Psychol*. 42 145–150. 10.1016/j.copsyc.2021.10.004 34794101

[B78] Stone B. M. (2021). The ethical use of fit indices in structural equation modeling: Recommendations for psychologists. *Front. Psychol*. 12:783226. 10.3389/fpsyg.2021.783226 34887821 PMC8650002

[B79] Tuominen-Soini H. Salmela-Aro K. (2014). Schoolwork engagement and burnout among Finnish high school students and young adults: Profiles, progressions, and educational outcomes. *Dev. Psychol*. 50 649–662. 10.1037/a0033898 23895174

[B80] Vallerand R. J. Pelletier L. G. Blais M. R. Briere N. M. Senecal C. Vallieres E. F. (1992). The academic motivation scale: A measure of intrinsic, extrinsic, and amotivation in education. *Educ. Psychol. Meas.* 52 1003–1017. 10.1177/0013164492052004025

[B81] Veijonaho S. Hietajärvi L. Ojala M. Salmela-Aro K. (2025). From distress to action? – A three-wave longitudinal study of climate change distress, pro-environmental behavior, and coping strategies among finnish adolescents. *J. Environ. Psychol.* 105:102676. 10.1016/J.JENVP.2025.102676

[B82] Veijonaho S. Ojala M. Hietajärvi L. Salmela-Aro K. (2024). Profiles of climate change distress and climate denialism during adolescence: A two-cohort longitudinal study. *Int. J. Behav. Dev.* 48 103–112. 10.1177/01650254231205251

[B83] Vercammen A. Oswald T. Lawrance E. (2023). Psycho-social factors associated with climate distress, hope and behavioural intentions in young UK residents. *PLoS Glob. Public Health* 3:e0001938. 10.1371/journal.pgph.0001938 37610987 PMC10446227

[B84] Wigfield A. Eccles J. S. (2000). Expectancy-value theory of achievement motivation. *Contemp. Educ. Psychol*. 25 68–81. 10.1006/ceps.1999.1015 10620382

[B85] Wu J. Snell G. Samji H. (2020). Climate anxiety in young people: A call to action. *Lancet Planet. Health* 4 e435–e436. 10.1016/S2542-5196(20)30223-0 32918865

[B86] Zurlo M. C. Vallone F. Mordente N. N. (2023). Assessing motivation in university students: Factor structure and psychometric properties of the Italian version of the academic motivation scale-college (AMS-C). *TPM* 30 43–61. 10.4473/TPM30.1.4

