# Supplementary material for: University students in the era of climate change: climate distress is associated with academic burnout and engagement through academic amotivation
Source: Front Psychol. 2026 Aug 26;17:1878132. doi: 10.3389/fpsyg.2026.1878132 (PMC13553446; doi:10.3389/fpsyg.2026.1878132)
Supplement: Supplementary file 1 [file Supplementary_file_1.docx]

**Supplementary Materials**

**Appendix A – Item adopted from the Italian version of the Academic Motivation Scale – College (AMS-C; Zurlo et al., 2023).**

Below is the selection of items adopted in the present study relating to the Italian version of the Academic Motivation Scale – College (AMS-C; Zurlo et al., 2023). The original scale is available in Open Access under CC BY-NC-ND 4.0 International License.

**Introjected Motivation Dimension**

1. Because I want to show myself that I can succeed in my studies

[*Perché voglio dimostrare a me stesso che posso avere successo nei miei studi*]

2. For the pleasure that I experience while I am surpassing myself in one of my personal accomplishments

[*Per il piacere che provo mentre cerco di superare me stesso raggiungendo nuovi traguardi personali*]

3. To show myself that I am an intelligent person

[*Per dimostrare a me stesso che sono una persona intelligente*]

**Intrinsic Motivation Dimension**

4. For the “high” feeling that I experience while reading about various interesting subjects

[*Per la forte attrazione che provo quando leggo vari argomenti interessanti*]

5. For the pleasure that I experience when I read interesting authors

[*Per il piacere che provo quando leggo autori interessanti*]

6. For the pleasure that I experience in broadening my knowledge about subjects that appeal to me

[*Per il piacere che provo nell’ampliare le mie conoscenze su argomenti che mi piacciono*]

**Extrinsic Motivation Dimension**

7. In order to obtain a more prestigious job later on

[*Per ottenere un lavoro più prestigioso in futuro*]

8. In order to have a better salary later on

[*Per avere un salario migliore in futuro*]

9. Because I want to have “the good life” later on

[*Perché voglio avere una bella vita in futuro*]

**Amotivation Dimension**

10. I don’t know; I can’t understand what I am doing in school

[*Non lo so; non riesco a capire quello che ci faccio all’università*]

11. I can’t see why I go to college and, frankly, I couldn’t care less

[*Francamente non so perché vado all’università. Non me ne importa nulla*]

12. I once had good reasons for going to college; however, now I wonder whether I should continue

[*Una volta avevo buone ragioni per andare all’università; tuttavia, ora mi chiedo se sia il caso di continuare*]

**Reference:**

Zurlo, M. C., Vallone, F., and Mordente, N. N. (2023). Assessing motivation in university students: factor structure and psychometric properties of the Italian version of the academic motivation scale-college (AMS-C). *TPM* 30, 43-61. doi: 10.4473/TPM30.1.4
